# Editorial: General anesthesia: from theory to experiments

**DOI:** 10.3389/fnsys.2015.00105

**Published:** 2015-07-22

**Authors:** Axel Hutt, Anthony G. Hudetz

**Affiliations:** ^1^Team Neurosys, INRIAVillers-les-Nancy, France; ^2^Team Neurosys, Centre National de la Recherche Scientifique, LORIA, UMR No. 7503Villers-les-Nancy, France; ^3^Team Neurosys, University of Lorraine, LORIA, UMR No. 7503Villers-les-Nancy, France; ^4^Department of Anesthesiology, Medical College of WisconsinMilwaukee, WI, USA

**Keywords:** consciousness, functional connectivity, coherence, EEG, fMRI, burst-suppression

General anesthesia is a standard and safe medical procedure performed in thousands of patients every day; therefore, it may come to many as a surprise that the ultimate neurobiological mechanisms responsible of the anesthetics' beneficial effect—that they suppress the patient's conscious awareness, is far from understood. One reason for this lack of understanding is that complex interactions of neurons occur on different spatial and temporal scales and the action of anesthetics on neural populations is poorly understood. Consequently, there is a need to bridge the knowledge of how anesthetic agents act on synaptic receptors on a microscopic scale to macroscopic changes in neuronal population activity as well as to higher order integrative processes that are more directly linked to the state of consciousness. The 10 contributions compiled in this research topic (ebook) intend to help solving this problem by exploring and adding to the current state of knowledge at various levels of brain complexity.

The first chapter by Mashour (2014) presents an authoritative review of the currently most influential theory of how chemically diverse general anesthetics on higher order processes may disrupt consciousness. The formerly favored “bottom-up” mechanisms of anesthetic action focusing on subcortical arousal centers and ascending thalamocortical information transfer are contrasted with the more recent cortical “top-down” explanations that are inherent to conscious perception and appear to be the preferential target of anesthetic modulation. Substantial electrophysiological and neuroimaging evidence from animal and human investigations supports the top-down mechanisms as a causally sufficient explanation for anesthetic-induced unconsciousness.

Raz et al. (2014) provides new support to this idea from their study of the effect of isoflurane on top-down vs. bottom-up neuronal pathways in rat auditory cortex during sensory and thalamic stimulation. By laminar recordings of local field potentials in the auditory cortex *in vivo*, they show that at hypnotic dose of isoflurane, bottom-up responses to auditory tone stimuli are enhanced, whereas top-down responses to visual flash stimuli are reduced. Consistent results were obtained in rodent brain slices, where cross-modal cortico-cortical descending pathways were suppressed far greater than specific thalamo-cortical afferents, supporting the preferential disruption of top-down connectivity at an anesthetic concentration associated with unconsciousness.

In the next chapter, Blain-Moraes et al. (2014) moves this idea to humans by demonstrating in surgical patients that anesthetic-invariant electroencephalographic effects occur in cortical top-down connectivity. Specifically, ketamine, a primarily non-GABAergic anesthetic drug, is found to suppress fronto-parietal functional and directional connectivity (measured by coherence and phase lag index), similar to that produced by propofol, a primarily GABAergic drug. Unlike propofol however, ketamine fails to augment frontal alpha power and coherence. The measured connectivity changes in the alpha band are therefore consistent markers of unconsciousness induced by both GABAergic and non-GABAergic anesthetics.

Moving on to deeper anesthetic levels, Kenny et al. (2014) explore anesthetic agent-dependent effects on burst-suppression patterns in rats. Burst suppression is a stereotypic pattern of alternating periods of electroencephalographic activity and inactivity that occurs in pathological states and in deep anesthesia, well beyond the threshold for loss of consciousness. After reviewing the presumed mechanism of generation, methods of quantification, and clinical application of burst-suppression, the authors demonstrate significant differences in the duration, amplitude, and power of burst-suppression patterns induced by two common general anesthetics, sevoflurane, and propofol suggesting that the neuronal circuits involved in burst-suppression generation may differ among different anesthetics.

By virtue of the similarity of anesthetic-induced loss of consciousness to the one experienced in sleep, anesthetic, and sleep research typically borrow analysis methods and neural processing concepts from each other. MacIver and Bland (2014) have compared frontal cortical and hippocampal micro-EEG signals under isoflurane anesthesia and during sleep by a chaos analysis. The shape of chaotic attractors of cortical frontal micro-EEG flattens in the anesthetic state compared to the awake state. In addition, delta-activity under isoflurane anesthesia exhibits a different chaotic attractor shape than NREM-sleep frontal EEG. The chaotic analysis demonstrates the power of nonlinear analysis methods revealing signal features beyond the frequency content.

In addition to the analysis of experimental data, theoretical models might provide deeper insight into the underlying neural mechanisms during general anesthesia. Hight et al. (2014) have modeled experimental EEG data obtained in individual human subjects during emergence from anesthesia to wakefulness by a neurophysiological model. This projection allows one to visualize the signal evolution in time and indicates differences between subjects. The study reveals an archetypical emergence pattern and non-archetypical evolution patterns which are all different from the archetypical emergence patterns. In addition to this classification, for all patients, a general neuronal hyperpolarization (increased resting membrane conductivity and reduced excitatory connection strength) appears to precede the return to consciousness.

The work by Hudetz et al. (2014) focuses on large-scale mechanisms combining human fMRI data and computer simulation to explore the diversity of brain connectivity patterns as a determinant of the state of consciousness. Implementing a spin-glass model with site interactions probabilistically defined by long-range functional connectivity, they predict the formation of metastable brain states whose repertoire is a function of cortical activation. The state repertoire is maximal at an optimal activation level corresponding to the conscious state. It is diminished in anesthesia (low activation) and seizure (high activation) suggesting a common mechanism for unconsciousness through a reduction of the brain's state repertoire.

To understand brain network interactions before and after loss of consciousness, the study of phase coherence provides valuable insights. Wang et al. (2014) have combined a detailed phase coherence study of experimental scalp EEG in the sub-delta frequency range with a theoretical model study. They have revealed a drop of phase coherence between electrode pairs in frontal, occipital, and fronto-occipital pairs. Conversely, the authors have revealed increased phase coherence between temporal and frontal, temporal, and occipital regions and temporal regions on left and right side. Theoretical model results confirm these findings and indicate a compensatory mechanism of sub-delta activity between a fronto-occipital and temporal region subsystem.

Anesthetic agents are known to affect various neural receptor types. They modify neural functions and inter-neuron interactions on the microscopic scale, consequently neural populations and eventually macroscopic electromagnetic activity, such as EEG/MEG/fMRI, and the behavior of subjects. To understand this bridge over multiple scales, Hashemi et al. (2014) have worked out a theoretical thalamo-cortical model demonstrating how GABAergic extra-synaptic receptors on a microscopic scale affect EEG on the macroscopic scale under propofol anesthesia. It turns out that cortical and thalamic anesthetic action on GABAergic extra-synaptic receptors contribute to the generation of delta-activity pointing out their importance.

In addition to action on extra-synaptic receptors, some anesthetics are known to desensitize synaptic receptors and may deplete synaptic vesicles. Bojak et al. (2014) hypothesize that these anesthetic actions contribute primarily to burst suppression. In a theoretical spatially extended cortical model assuming isoflurane action, they reveal spatially heterogeneous burst suppression patterns propagating in the cortex. This work provides an additional possible mechanism for burst suppression.

## Conflict of interest statement

The authors declare that the research was conducted in the absence of any commercial or financial relationships that could be construed as a potential conflict of interest.
